# Measuring Health-Related Quality of Life in Strabismus: A Modification of the Adult Strabismus-20 (AS-20) Questionnaire Using Rasch Analysis

**DOI:** 10.1371/journal.pone.0127064

**Published:** 2015-05-26

**Authors:** Vijaya K. Gothwal, Seelam Bharani, Ramesh Kekunnaya, PreetiPatil Chhablani, Virender Sachdeva, Niranjan K. Pehere, Asa Narasaiah, Rekha Gunturu

**Affiliations:** 1 Meera and L B Deshpande Centre for Sight Enhancement, L V Prasad Eye Institute, KallamAnji Reddy campus, Hyderabad, India; 2 Jasti V Ramanamma Children’s Eye Care Centre, L V Prasad Eye Institute, Hyderabad, India; 3 Nimmagadda Prasad Children’s Eye Care Centre, L V Prasad Eye Institute, GMR Varalakshmi Campus, Vishakapatnam, India; 4 The David Brown Children’s Eye Care Centre, KodeVenkatadriChowdary Campus, Vijayawada, India; Medical College of Soochow University, CHINA

## Abstract

**Purpose:**

To evaluate the psychometric properties of the Adult Strabismus-20 (AS-20)- a health-related quality of life (HRQoL) questionnaire in adults with strabismus, and if flawed, to revise the AS-20 and its subscales creating valid measurement scales.

**Methods:**

584 adults (meanage, 27.5 years) with strabismus were recruited from an outpatient clinic at a South Indian tertiary eye care centre and were administered the AS-20 questionnaire.The AS-20 was translated and back translated into two Indian languages. The AS-20 and its two 10-item subscales – ‘psychosocial’ and ‘function’were assessed separately for fit to the Rasch model, including an assessment of the rating scale, unidimensionality (by principal components analysis), measurement precision by person separation reliability, PSR, targeting, and differential item functioning (DIF; notable > 1.0 logits).

**Results:**

Response categories were not used as intended, thereby, required re-organization and reducing their number from 5 to 3. The AS-20 had adequate measurement precision (PSR = 0.87) but lacked unidimensionality; however, deletion of the six multi-dimensionality causing items and an additional three misfitting items resulted in 11-item unidimensional questionnaire (AS-11). Two items failed to satisfy the model expectations in the ‘psychosocial’ subscale and were deleted – resulting in an 8-item unidimensional scale with adequate PSR (0.81) and targeting (0.23 logits). One item misfit in the ‘function’ subscale and was deleted—resulting in a 9 item Rasch-revised unidimensional subscale with acceptable PSR (0.80) and targeting (0.97 logits).None of the items displayed notable DIF by age, gender and level of education.

**Conclusions:**

The AS-11 and its two Rasch-revised subscales – 8-item psychosocial and 9-item function subscale may be more appropriate than the original AS-20 and its two 10-item subscales for use as unidimensional measures of HRQoL in adults with strabismus in India. Further work is required to establish the validity of the revised rating scale.

## Introduction

Strabismus in adults is a common problem,and is present in approximately 4% of the population [1]. It is associated not only with functional effects, but also has adverse effects on a patient’s quality of life (QoL) [2].Moreover, the impact of strabismus on QoL has been reported to be greater than that caused by chronic sight-threatening ocular conditions such as diabetic retinopathy, but comparable to that seen with macular degeneration or following a mild cerebrovascular accident[3].Over the last two decades, there have been several reports of the negative psychosocial effects of strabismus in adults with patients reporting all aspects of their lives being affected by manifest strabismus—self-image, self-esteem, confidence, job prospects, relationships, education and participation in sports [4–10].It has also been reported that these problems tend to getaggravated as they grow older and strabismus size increases[7]. Furthermore, a higher proportion of strabismic adults (41.3%) have been shown to develop mental health problems as compared tothe controls (30.7%) [11–13].

Several HRQoL instruments have been used to evaluate the aforementioned effects of strabismus in adults [3, 4, 6, 7, 9, 14, 15]. Health-related QoL (HRQoL) is a concept that incorporates physical, functional and emotional status, and social functioning [16]. Although HRQoL can be assessed using either generic or condition-specific instruments, there are concerns that the former may not focus adequately on problems specific to a particular disease and that they may simultaneously measure the impact of comorbid disease. By comparison, condition-specific instruments have advantages in that these are more responsive as they address concerns that are important to a particular patient population [17–19].The recently developed Adult Strabismus-20 (AS-20) questionnaire by Hatt et al. is one such instrument developed specifically to assess the HRQoL in adults with strabismus [15].It consists of 20 items divided over 2 subscales with 10 items each—the ‘psychosocial’ and ‘function’ subscale. The AS-20 questionnaire has been shown to be highly responsive to changes in ocular alignment and symptoms after strabismus surgery [20].The overall high performance of the original AS-20 questionnaire such as its reliability, validity and responsiveness to change associated with surgical treatment dictates that its popularity will be further enhanced among the ophthalmic community[20]. Consequently, there have been recent reports of its evaluation and use in China [21–23],and in India [24].Using the classical test theory (CTT) approach, the psychometric performance (reliability, validity) of the Chinese version of the AS-20 was reported to be consistent with the original AS-20 [21]. Nonetheless, its responsiveness hasn’t been reported as yet. Unlike the Chinese study, the psychometric properties of the AS-20 in the Indian population haven’t been reported.

Like most instruments in health care, the AS-20 questionnaire was developed using traditional psychometric approaches (CTT). It has been shown to be a reliable (overall Cronbach alpha = 0.94 and 0.91 in American and Chinese populations respectively) and a valid measure of HRQoLamong strabismic adults [15, 21, 25].However, there are limitations to CTT methods, such as assumptions of interval data level, normal distribution of responses and that each item contributes equally to the construct under measurement. Modern psychometric methods, such as Rasch analysis provides superior scale information to CTT methods. Rasch analyses enable examination of scale functioning at item and overall levels, scale dimensionality, and category response functioning [26–29]. Given these advantages of Rasch models, Leske et al. applied it to the AS-20 questionnaire in an American adult population with strabismus [30].They assessed several psychometric characteristics such as unidimensionality, targeting, item difficulty, separation, behaviour of rating scale and differential item functioning (these terms have been explained in detail later). Leske et al. reported deficiencies in the performance of the AS-20,such as the presence of misfitting items, dysfunctional rating scale, poor targeting for the interaction subscale, and poor measurement precision (reliability) for both the interaction and general subscale.However, targeting is sample dependent[31], and therefore makes it important that the AS-20 questionnaire is tested using Rasch analysis in other strabismic populations. Based on their results of Rasch analysis, the investigators suggested refinements to the AS-20 questionnaire: subscale restructuring, reduction of items within the predefined subscales, and reduction in the response options of the general function subscale from 5 to 4. The revised version consists of 4 new subscales—self-perception,interaction (both belong to psychosocial subscale), reading, and general function (both belong to thefunction subscale).

Information about the performance of the AS-20 questionnaire using Rasch analysis in different populations is non-existent. More importantly, there is a need to explore the performance of HRQoL questionnaires in populations (e.g. Indian) different to those for whom these were originally developed for (American). This information is vital because populations could differ in terms of clinical and demographic characteristics, such as, the amount of deviation, age, and gender. As it pertains to the effects of strabismus on HRQoL, some of the cultural differencesthat should be considered between the Indian and American populations include, firstly, the psychological effects of strabismus on the self-esteem (lack of discrepancy between one’s self image and actual self image [32]).It is believed that Asians report lower levels of self-esteem than the Americans [33].This difference may be partly explained by the fact that East Asian cultures emphasize the importance of group [34], and so socialization practices in these cultures might not emphasize the expression of personal high self-esteem because such expressions could be viewed as opposing the culturally valued attributes that promote group harmony [35]. On the other hand, the expression of high self-esteem in more individualistic cultures (e.g. American) might be encouraged given the cultural value placed on freedom and individual rights [36]. Such culturally informed orientations may affect the way that participants respond to questionnaires, particularly those that emphasize self-values. This tendency might manifest as a consistent response style that reflects a reluctance to use the extreme ends of scales for individuals from collectivist cultures such as East Asians [37]. People from such cultures may feel that choosing the extreme end of the scale would cause them to stand out from the group. Secondly, the difference of individualistic versus collectivist culture between the Americans and Indians in terms of social desirability. In a study, Middleton and Jones showed that social desirability is of greater influence in collectivistic cultures (e.g. Indian) than in individualistic cultures (e.g, American) [38]. Taken together, thedifferencesbetween the cultures interms of self-esteem and social desirability could have an influence on the choice of response options by the participants.

Given this background, the aim of this study was to translate and validate the Indian version of the AS-20 questionnaire in an Indian strabismic population, which is different to American in the amount of deviation, demographically and culturally.

## Patients and Methods

### AS-20 Questionnaire

The AS-20 is a freely available questionnaire consisting of 20 items divided into 2 subscales—psychosocial and function, with 10 items each (Table 1). Participants rate each item on a 5-point Likert scale with response categories that include “never (0)”,“rarely (1)”, “sometimes (2)”, “often (3)”, “always (4)”.The psychosocial subscale score is calculated as a mean of items 1 to 10. The function subscale score is calculated as a mean of items 11 to 20. In the present study, higher psychosocial and function subscale scores indicate worse HRQoL. Given that it was developed in English we translated it into two local languages (Hindi and Telugu) using standard forward-backward translation accepted procedures.For each language version, briefly, this included two forward independent translations (by two bilingual experts) into the target language followed by reconciliation to prepare a single version. This was then back translated by an independent expert into English and was assessed for any discrepancies from the source language. Any discrepancies were resolved by consensus of the group, and the pilot version was developed that was pre-tested in 5 representative strabismic patients (illiterate -1, high school-2, graduation and beyond -2 and, male—3, female—2). Following this testing the final version was developed and used in the main study.

**Table 1:** Item content of the Adult Strabismus Questionnaire-20*.

**Item No.**	**Item Description (Psychosocial subscale)**
1	I worry about what people will think about my eyes
2	I feel that people are thinking about my eyes even when they don’t say anything
3	I feel uncomfortable when people are looking at me because of my eyes
4	I wonder what people are thinking when they are looking at me because of my eyes
5	People don’t give me opportunities because of my eyes
6	I am self conscious about my eyes
7	People avoid looking at me because of my eyes
8	I feel inferior to others because of my eyes
9	People react differently to me because of my eyes
10	I find it hard to initiate contact with people I don’t know because of my eyes
	**Item Description (Function subscale)**
11	I cover or close one eye to see things better
12	I avoid reading because of my eyes
13	I stop doing things because my eyes make it difficult to concentrate
14	I have problems with depth perception
15	My eyes feel strained
16	I have problems reading because of my eye condition
17	I feel stressed because of my eyes
18	I worry about my eyes
19	I can’t enjoy my hobbies because of my eyes
20	I need to take frequent breaks when reading because of my eyes

Response categories of items 1–20 include: never, rarely, sometimes, often, always

*Formatted questionnaire available for download at www.pedig.net

### Participants

Participants were patients with manifest strabismus (of > 3 months duration)recruited from the outpatient clinics of the L V Prasad Eye Institute (LVPEI) at any of its 3 campuses across Hyderabad, Vishakapatnam and Vijayawada located in two neighbouring Indian states (Telangana and Andhra Pradesh).

We included participants aged ≥ 18 years, spoke English, Telugu or Hindi, had manifest strabismus, and no severe cognitive impairment. We excluded patients with facial dysmorphism or myasthenia gravis as well as those who were unable to converse in English, Telugu or Hindi.Research assistants explained the nature of the study to the participants and those who agreed signed a consent form following which they were administered the AS-20 questionnaire. We invited a total of 594 adults with strabismus to participate in the study (details are provided in S1 Datasheet). However, a few potential participants (n = 10) refused to participate for lack of time, yielding a response rate of 98.3% (n = 584). Although this number was too small, a formal analysis revealed that their demographic characteristics did not substantially differ from those included. While a majority (n = 514, 88%) self-administered the questionnaire, trained interviewers administered it face-to-face in a quiet room to the remaining participants. Interviewers attended a half-day training workshop conducted by experienced interviewers from LVPEI during which they were familiarized with the principles of questionnaire administration and supervised in the conduct of practice interviews prior to the start of the study. Demographic and clinical data of the 584 adults with strabismus who completed the AS-20 questionnaire are summarized in Table 2.

**Table 2:** Characteristics of the 584 participants who completed the Adult Strabismus-20 Questionnaire.

Characteristic	Result
Age (years)	
Mean ± SD	27.5 ± 9.4
Range	18–75
Gender, n (%)	
Male	362 (62)
Education level, n (%)	
Illiterate	20 (3)
Upto high school	196 (34)
Graduation and beyond	368 (63)
Location of residence, n (%)	
Urban	424 (73)
Marital status, n (%)	
Single	408 (70)
Married	172 (29)
Divorced/Widowed	4 (1)
Types of strabismus	
Esotropia	100 (17)
Exotropia	484 (83)
Deviation size, prism dioptres, n (%)	
≤ 25	152 (26)
> 25	432 (74)
Etiology of strabismus, n (%)^Ϯ^	
Childhood/Idiopathic	426 (75)
Neurogenic	22 (4)
Mechanical	43 (7)
Sensory	78 (14)
Visual acuity in better eye (logMAR)	
Mean ± SD	0.03 ± 0.12

^Ϯ^ Data not available for 15 patients

### Ethics Statement

We obtained ethical approval of the study from the Ethics Committee for Human Research at LVPEI, Hyderabad, India and all consenting participants provided written informed consent. The study was conducted in accordance with the tenets of the Declaration of Helsinki.

### Rasch analysis

We conducted Rasch analysis [39] using the Andrich rating scale model [40] with Winsteps software, version 3.68.0 [41].Although the AS-20 questionnaire was originally designed to have 2 subscales—psychosocial and function (implying the use of 2 separate subscale scores) by its developers [15],there has been evidence of use of total or overall AS-20 raw score in the literature [20, 22, 24].Nonetheless, in line with the recommendations of the developers of AS-20 questionnaire, Leske et al. analysed the two subscales separately in their Rasch analysis of the AS-20 in the American population and found each of these scales to in turn contain 2 subscales within them [30].Similar to Leske et al., we analysed the two AS-20 subscales separately using Rasch analysis.We used Rasch analysis to determine whether the overall subscale scores are valid and reliable, and possess adequatemeasurement characteristics. If we found flaws in the subscales, we attempted to create re-engineered versions of these.

The Rasch measurement model has been described elegantly by Massof [42].Furthermore the various steps involved in Rasch analysis have been described by us earlier. Therefore we describe these in brief only here. We used four fundamental indicators to evaluate questionnaire quality. These included (i) fit, or the extent that items in the AS-20 and its subscales measure a single construct (i.e., unidimensionality), (ii) item endorsability, (iii) targeting, or the extent to which the set of items is of appropriate endorsability for the level of participant’s HRQoL, and (iv)measurement precision using person separation reliability (PSR), or the extent to which the items distinguish distinctlevels of HRQoLamong the participants.

As a first step in the analysis, we used the category probability curves to scrutinize the rating scale functioning. For a well-functioning rating scale, each category should be represented by a curve with a distinct peak. In addition such a performance will manifest as monotonic increase in both averagemeasures and thresholds across rating scale categories. Disordered thresholds indicate a serious problem with a scale that must be addressed by collapsing the rating scale categories. If there were disordered thresholds then we considered collapsing the rating scale categories.

Next, we used Rasch fit statistics in combination with principal components analysis (PCA) of residuals to test the dimensionality of each of the subscales. As the Rasch model is probabilistic, some amount of deviation in scores is expected. This deviation in expected versus observed scores is captured by fit statistics (i.e. infit mean square, or MnSq). The ideal value of InfitMnSq is 1.0 (indicates no deviation). In accordance with the literature, we used infitMnSq values between 0.7 and 1.3 as an indicator of acceptable fit [28, 45, 46].We considered items outside this range as misfits [47] and deleted them in an iterative manner, beginning with deletion of the most misfitting item until all the remaining items fit well. Given that fit statistics alone are insufficient to confirm unidimensionality we used PCA of residuals for this purpose [48]. Common criteria for unidimensionality assessment using PCA of residuals is that at least 60% of the variance should be explained by the first dimension (i.e. each subscale) and the eigenvalue for the second largest dimension should be <2.0 [26, 43, 49, 50].

We investigated targeting (measure of how well the item endorsability matched with person’s HRQoL) by visualizing the person-item map and comparing the means of the items and person measures. Good targeting of the item difficulty to person ability is important to minimize floor and ceiling effects and to ensure that the instrument or subscale is measuring HRQoL in a way that is appropriate for the population’s HRQoL level.

We assessed measurement precision using PSR and the reliability coefficient ranges from 0 to 1: coefficients 0.80 are considered as good and 0.90 as excellent [51–53].We considered an adequate PSR (0.8 and above) as a prerequisite for the subscale to be termed as a measure.

If the data fit the Rasch model, Rasch analysis allows detection of differences in item difficulties between different groups within a sample. This is referred to as the differential item functioning (DIF). We selected the DIF variables a *priori* in the present study. DIF was investigated for age (split at median; ≤25 vs>25 years), gender, location of residence (urban vs rural), and education (upto high school vs graduation and beyond).DIF was considered to be absent if it was less than 0.50 logits, and minimal (but probably inconsequential) if between 0.50 to 1.0 logits and notable if >1.0 logits [54, 55].Descriptive statistics were analyzed using SPSS software (version 15.0).

## Results

### Psychometric properties of the AS-20 and its subscales

#### Assessment of response categories

**Fig 1:**
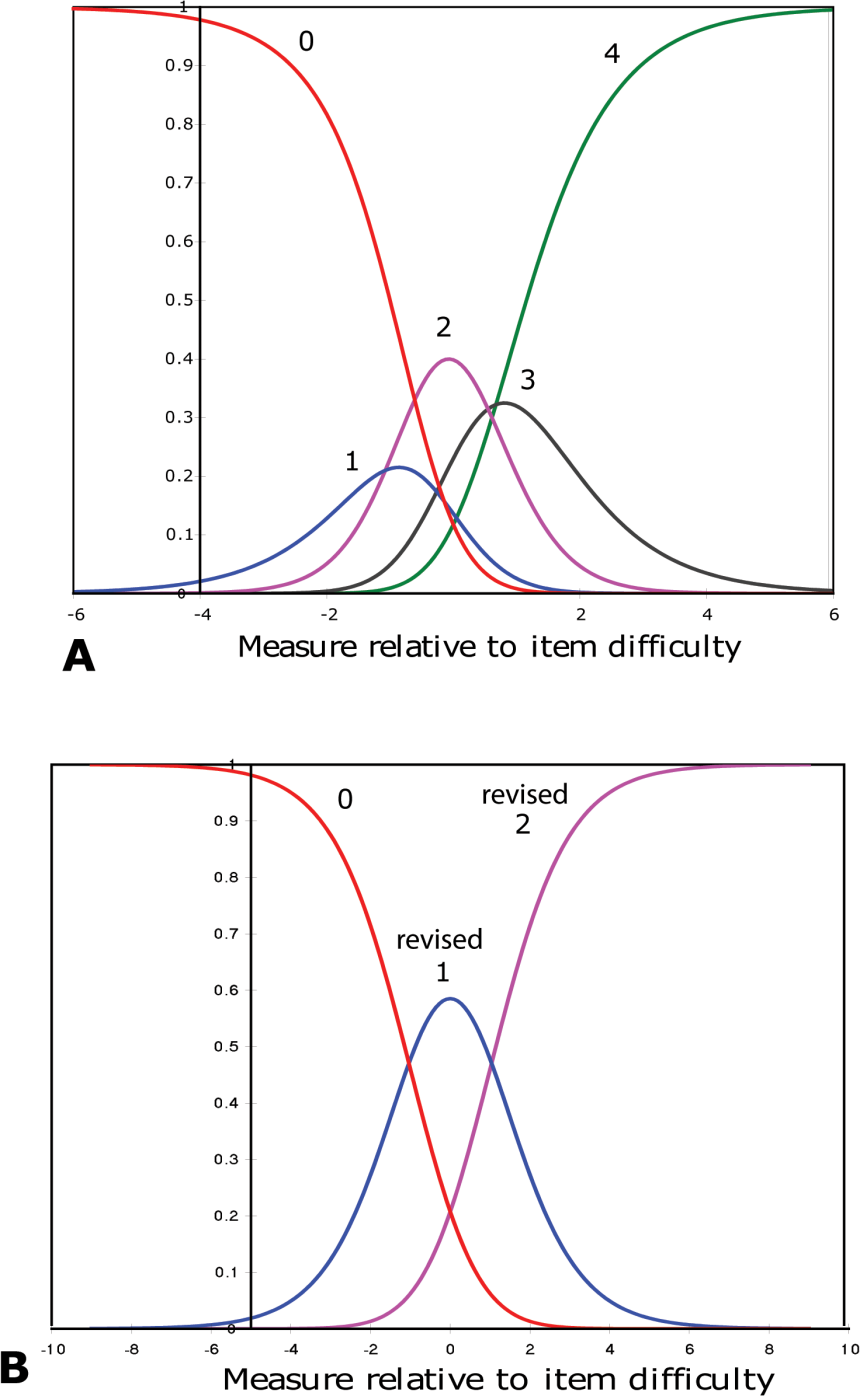
Rasch model category probability curves for all items together in the AS-20 showing the likelihood that a participant with a particular coping ability will select a category. **A.** The scale (*x*-axis) from +6 to -6 symbolizes the latent trait of health-related quality of life and the *y*-axis represents the probability of category being selected. Response categories: 0 “never”, 1 “rarely”, 2 “sometimes”, 3 “often” and 4 “always”. For any given point along this scale, the category most likely to be chosen by a participant is shown by the category curve with the highest probability. At no point, was category 1 the most likely to be chosen and appears to be interchangeable with categories 0 and 2, resulting in disordered thresholds. **B**. Ordered thresholds following category re-organization and reducing the number of categories from 5 to 3 for the category probability curves seen in Fig A. Thresholds represent boundaries along the scale where the probability of a response category being chosen changes from one to the next.

Participants did not use the response categories as intended.Categories that perform adequately should have ordered structure calibration thresholds. This indicates that each category has a distinct probability of being chosen more than any other category for a particular item of endorsability. However, this pattern is lacking in our case. Fig 1A shows the category probability curves which illustrate the range of HRQoL for which each of the 5 response categories are most likely to be chosen. It can be seen that at no point on the logit scale is the probability of responding to category 1 “rarely” greater than the probability of responding to category 0 (“never”) or category 2 (“sometimes”). Therefore, this response category did not function as expected, thereby, resulting in disordered thresholds. This is further illustrated in Table 3 by the lack of monotonic increase in the step calibration values. We have the option to combine this category with either 0 or 2. However, we chose the category combination that provided the best targeting and PSR. Consequently, we rescored the response categories by collapsing category 1 and 2 into a single new category “rarely/sometimes” (01123); however disordering continues as before, albeit between different thresholdsnecessitating combining revised categories 2 and 3 into a single new category “often/always” and following this step the thresholds demonstrate an ordered behavior (Fig 1B, Table 3). Thus, category re-organization reduced the number of categories from 5 to 3 (Table 3). It can be further seen from Table 3 that there is a monotonic increase in the step calibration valuesafter final category re-organization (3 categories).

**Table 3:** Adult Strabismus-20 questionnaire (AS-20) before (left, 5 categories) and after (centre, 4 categories, and right, 3 categories) the collapsing procedure.

Adult Strabismus -20											
5 categories				4 categories				3 categories			
Cat. label	Obs. count (%)	Avg. meas.	Step calib.	Cat. label	Obs. count (%)	Avg. meas.	Step calib.	Cat. label	Obs. count (%)	Avg. meas.	Step calib.
0	45	-1.28	None	0	45	-1.85	None	0	45	-1.68	None^Ϯ^
1	13	-0.69	0.19	1+2	32	-1.74	-1.00	1+2	32	-0.28	-0.70
2	19	-0.32	-0.92	-	-	-	-	-	-	-	-
3	11	0.13	-0.42	3	11	0.06	0.66	3+4	23	0.94	0.70
4	12	0.48	0.31	4	12	0.59	0.35	-	-	-	-

Cat. label—Category label; Obs. Count: Observed count—number of times each response category was used across items and participants in percentage; Avg. measure: Average measure—mean ability of the participants getting a given score; Step calib.—Step calibrations or Rasch/Andrich thresholds correspond to the ability level at which adjacent scores are equally likely

^Ϯ^No threshold presented for category 0 because it is the lowest category.

#### Overall performance of Psychosocial subscale

**Fig 2:**
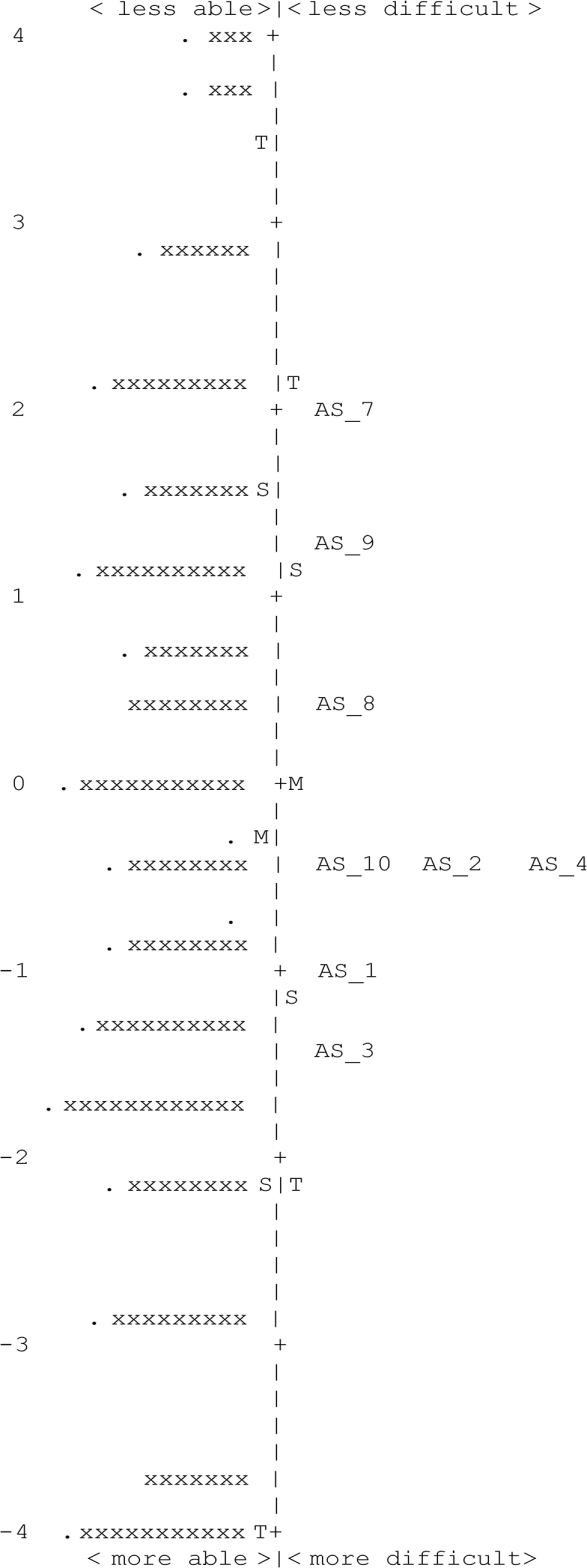
Person-item map for the Rasch-revised 8-item ‘psychosocial’ subscale of the AS-20 questionnaire. Participants are located on the left of the dashed line (represented by ‘x’) and participants with worse health-related quality of life are located at the top of the map. Items are on the right of the dashed line with those considered to be less difficult to endorse are located toward the top of the map. Each ‘x’ and “.” represent four and one participants respectively. Alongside each item is also indicated its original item number as in the AS-20 questionnaire. The complete description of items can be found in Table 1 in the text. M, mean; S, 1 SD from the mean; T, 2 SD from the mean.

Of the 10 items in this subscale, one item (‘I am self conscious about my eyes”) misfit (InfitMnSq, 1.76) necessitating its deletion. PCA of residuals indicated that 62.1% of the variance was explained by the first dimension and the unexplained variance explained by the first contrast was 1.7 eigenvalue units, indicating unidimensionality (Table 4).Person separation reliability was 0.82 (person separation = 2.14), and targeting of item endorsability to the participant’s HRQoL was 0.22 logits. We deleted the misfitting item following which another item misfit (“People don’t give me opportunities because of my eyes”) (InfitMnSq, 1.32). Following the deletion of the second item, all the remaining 8 items fit the Rasch model well. The person separation reliability was 0.81 (person separation = 2.06) and targeting of item endorsability to participant’s HRQoL was 0.23 logits (Fig 2). None of the items displayed notable DIF (Table 4).

**Table 4:** Overall performance of the AS-20 and its two subscales in adults with strabismus.

Parameter	Ideal values	Versions of AS-20 and its subscales					
		AS-20		Psychosocial		Function	
		Original	Revised*	Original	Revised*	Original	Revised*
Number of items	-	20	11	10	8	10	9
No. of misfitting items	0	2	0	2	0	1	0
Person separation	≥2.0	2.64	2.40	2.14	2.06	2.04	2.01
Reliability	≥0.80	0.87	0.85	0.82	0.81	0.81	0.80
Mean item location	0	0	0	0	0	0	0
Mean person location	0	0.64	0.31	0.22	0.23	0.98	0.97
Principal components analysis (eigenvalue)	≤2.0	3.4	1.8	1.7	1.7	1.7	1.7
Differential item functioning, DIF^Ϯ^ (Number of items with notable DIF, >1.0 logits)	0	0	0	0	0	0	0

AS-20—Adult strabismus-20 questionnaire

*Misfitting items were deleted iteratively and final revised versions are shown here. See text for details.

^Ϯ^Differential item functioning analyses was performed and tested across age, gender, location of residence and educational level.

#### Overall performance of Function subscale

**Fig 3:**
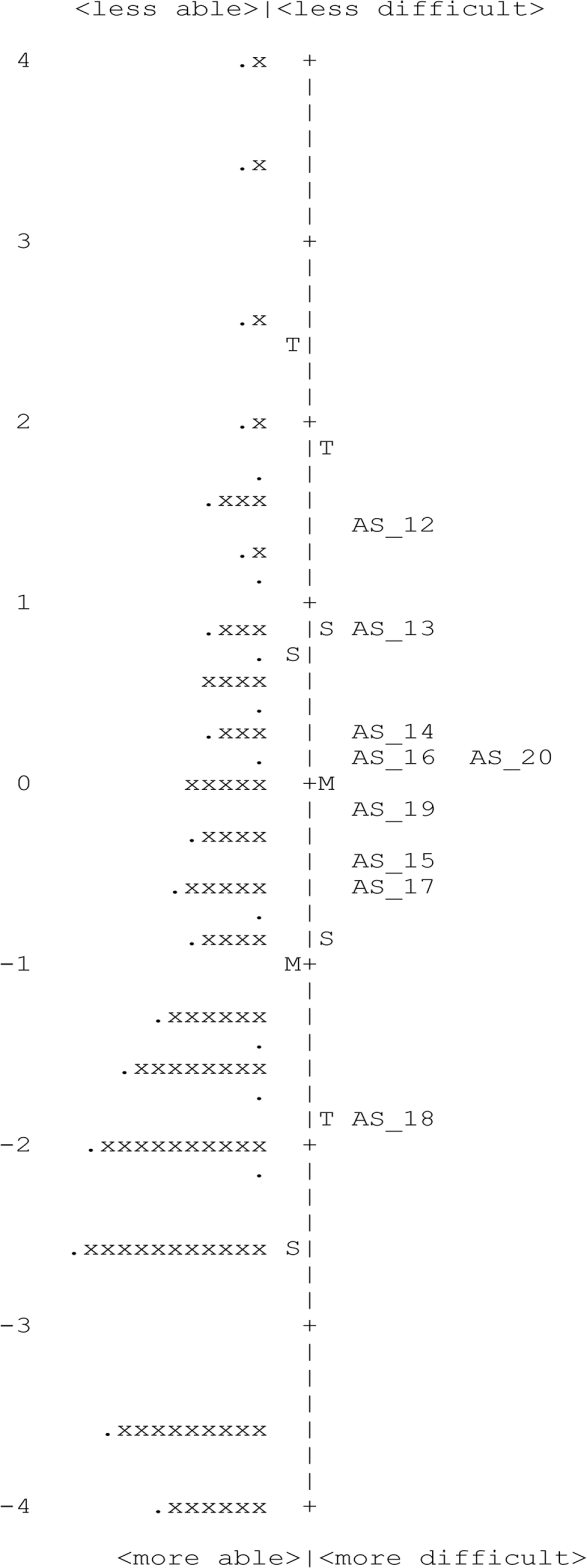
Person-item map for the Rasch-revised 9-item ‘function’ subscale of the AS-20 questionnaire. Participants are located on the left of the dashed line (represented by ‘x’) and participants with worse health-related quality of life are located at the top of the map. Items are on the right of the dashed line with those considered to be less difficult to endorse are located toward the top of the map. Each ‘x’ and “.” represent six and one participants respectively. Alongside each item is also indicated its original item number as in the AS-20 questionnaire. The complete description of items can be found in Table 1 in the text. M, mean; S, 1 SD from the mean; T, 2 SD from the mean.

Of the 10 items in this subscale, one item (‘I cover or close one eye to see things better’) misfit (InfitMnSq, 1.39) necessitating its removal. PCA of residuals indicated that 63.2% of the variance was explained by the first dimension and the unexplained variance explained by the first contrast was 1.7 eigenvalue units, indicating unidimensionality (Table 4). Person separation reliability was 0.81 (person separation = 2.04) and targeting of item endorsability to the participant’s HRQoL was 0.98 logits. We deleted the misfitting item following which all the remaining 9 items fit the Rasch model well. The person separation reliability was 0.80 (person separation = 2.01) and targeting of item endorsability to participant’s HRQoL was 0.97 logits (Fig 3). None of the items displayed notable DIF (Table 4).

### Raw score to Rasch measure conversion

Given that population samples vary, it is ideal to perform Rasch analysis on the data of a given study. However, other investigators may wish to use the AS-11and gain the interval scoring benefits of Rasch analysis, without performing Rasch analysis themselves. Therefore, we have provided a series of Excel (Microsoft, Redmond WA, USA) spreadsheets that convert ordinal category responses to Rasch measurement estimates. However it should be borne in mind that these conversions will only hold when the sample included is similar to that of ours. The sheets are available for two functional subscales—the 8 item psychosocial (see S2 Datasheet) and 9 item function subscale (see S3 Datasheet), and these can be downloaded from the journal’s website.

## Discussion

This study provides new insights into the measurement psychometric properties of the two subscales of AS-20 in adults with strabismus in an Indian population. Using Rasch analysis, the psychometric properties of the two original subscales of the AS-20 were partially confirmed; however, item deletion and rescaling were necessary to some degree for both the subscales.

Unidimensionality is an important prerequisite for summating any set of Likert-style items commonly seen in HRQoL instruments [56, 57], and therefore constitutes an important advantage if meaningful measurement is to be obtained[56, 58].Our results of Rasch analysis of the two subscales of the AS-20 indicate that each of them is largely a unidimensional measure of HRQoL in Indian adults with strabismus. This provides confidence that the summation of individual item scores to obtain a total “HRQoL” score is indeed provided by the subscales. The results of Rasch analysis also indicate that an interval level scoring system could be obtained for the subscales of AS-20 for this population. However, our results regarding the unidimensionality of the two AS-20 subscales are at variance with the findings of the previous Rasch analysis of the instrument by Leske et al.[30].They found both the subscales to lack unidimensionality given the presence of a second dimension and recommended formation of four new subscales (albeit two of these being dysfunctional given their low measurement precision). In their study, 4 items (items 1, 2, 3, 4)wereassociated with a second dimension for the psychosocial subscale.They argued that while these items belong to the overall psychosocial (i.e., involving aspects of both social and psychological behaviour) subscale, they involve a component of self-perception (i.e., the idea that you have about the kind of person you are). Similarly, they found 5 items (items 12, 13, 16, 19, 20) to be associated with a second dimension (reading function) for the function subscale. By comparison, we found the two AS-20 subscales to fulfill the criteria for unidimensionality in our population. Presumably, this indicates that the self-perception items are also contributing to the measurement of HRQoL in the strabismus patient in India. One could argue a loss of face validity, but the data clearly show the same latent trait is being tapped so these items should be retained in the psychosocial subscale, although only in this population. The variability of the results of Rasch analyses of AS-20 from two different strabismic populations suggests that the performance of the AS-20 may vary by the population characteristics and reinforces the need for clinicians and researchers to formally test the psychometric properties of the instruments they intend to use on different populations. More importantly, such variations in the psychometric properties of a HRQoL instrument render it impossible to compare HRQoL results across cultural groups, something which would be highly desirable. We speculate that the notable differencesin sample characteristics between the two studies may have been responsible for this variation in findings. Firstly, majority of our participants (75%) had childhood or idiopathic aetiology for the strabismus as compared to 44% in the previous study. Secondly, the median age of our participants was 25 years as compared to 52 years in the earlier study. Thirdly, most of our patients (83%) had exotropia as compared to only less than one-half (46%) with exotropiain the previous study. Thus, we believe that homogeneity of our sample may have been responsible for the unidimensionality of AS-20 subscales. In addition the cultural differences between the two populationswith strabismus (as noted in the Introduction) may underlie the differential patterns observed in our study and the previous study. Nonetheless, it is important to evaluate the dimensionality of the AS-20 subscales with a large sample with diverse characteristics so as to resolve the dilemma surrounding the dimensionality of the AS-20 subscales.

We found one item to be misfitting in each of the two AS-20 subscales and such misfit indicates that these itemswere not in tandem with the remaining items in the measurement of the underlying construct. In other words, participants responded to these items erratically perhaps because these items were not understood well, were ambiguous or measured a second dimension [29]. Of these, ambiguity appears the most likely in the present case because one of the items (‘I am self-conscious about my eyes’) pertains to self-consciousness,and is rather vague. Although the other item (‘I cover or close one eye to see things better’) pertains to function, it was perhaps considered a socially unacceptable behaviour among our sample. Removal of misfitting items usually improves the fit of the model[59].

Analysis of the rating scale of the AS-20 in our study indicated the need for category re-organization given the inconsistent endorsement of response categories. Response options ‘rarely’ and ‘sometimes’, and ‘often’ and ‘always’ were collapsed. Consequently, the revised rating scale consists of 3 response categories. The need for revision of response categories is not unique in our study given that reduction to a three-category rating scale has been found to be optimal in previous studies that have investigated response category utilization[52, 60]. The need for category re-organization was demonstrated in earlier Rasch analysis of AS-20 by Leske et al. study too, albeit for a single subscale. Issues with response categories can occur when the labeling of response categories is ambiguous or too many response options are included. Given that careful clinical judgement was employed during the development of the AS-20 questionnaire [15], it is unlikely that the five-point response options were ambiguous; rather, it is possible that items of the AS-20 hadtoo many response categories. Our findings are consistent with several studies that have undertaken Rasch analysis on other instruments which have found that participants are not always able to distinguish between finer increments in response options [26, 44, 61].However caution should be exercised in the interpretation of these findings as this is the first study to indicate the need to revise (shorten) the rating scale for AS-20 and these findings may be a function of the distinct study population, and not the instrument itself. Nonetheless, further testing in other validation samples would be necessary before proceeding with this modification.

The items in both the subscales of AS-20 were targeted well to the HRQoL of our participants indicating that the instrument is well equipped to assess the impact of strabismus on the HRQoL across the entire spectrum of the disorder in patients in India. By comparison, the earlier Rasch analysis of the AS-20 in the American population provided mixed results, with targeting being poor for one subscale, and acceptable for the remaining subscales [30].However, targeting is sample dependent so it is plausible that the results of targeting may not be replicable in other populations [31].

Both the subscales of the AS-20 showed good measurement precision in the Indian cohort indicating that each of these can reliably distinguish among several groups of participants and are sufficiently reliable for individual patient use [62].Although not directly comparable, this finding is at variance with the previous Rasch validation study of the AS-20 subscaleswherein there were mixed results regarding measurement precision of the four subscales [30]. Measurement precision is determined by the number of units into which the range is divided and is defined mainly by the number of items in the instrument. It is difficult to have satisfactory measurement precision with a small number of items [63]. Given this, it is not surprising that two of the four subscales in the earlier Rasch validation study of AS-20 lacked adequate precision. Greater measurement precision of the AS-20 subscales in our population coupled with less measurement error when evaluating HRQoL outcomes offers the benefit of smaller sample sizes needed to detect significant differences between groups[64].

We did not find notable DIF for any of the items in the present study indicating that the items of the AS-20 were behaving similarly across different sub-groups of participants in the Indian cohort. This finding is in accordance with that of the study by Leske et al. in the American population. The absence of notable DIF for the AS-20 subscales regarding the variables investigated is an important property of the instrument and helps further substantiate the construct validity of the AS-20 subscales. However, factors not investigated here may demonstrate DIF. Therefore, this warrants further study in specific target groups.

As our Rasch results are the first of their kind, they require validation in other samples before they can be used to recommend changes to the subscales of AS-20. It is possible that Rasch analysis of the AS-20 subscales in other strabismic populations would identify different items as requiring revision to those found in this study. Researchers using the AS-20 with strabismic adults, should therefore consider performing Rasch analysis on their data in order to ascertain the generalizability and robustness of the current findings. If, over time, multiple Rasch analyses identify the same items to misfit (e.g. item 6) then these could be rephrased, removed and/or rescored to improve psychometrics (perhaps in particular patient groups). Researchers would need to think about any such revisions that might impact the cross-group and study comparability of AS-20 research findings. Researchers using the AS-20 may also consider reanalyzing their data in line with current findings (i.e. using the revised rating scale and omitting misfitting items) and undertake exploratory analyses to understand the analytical impact of such psychometric modifications. It may be the case that the item rescoring and deletions indicated by the current study are insufficiently significant to substantively alter the results of any empirical analyses involving the AS-20[20, 65, 66]. This could potentially be an interesting avenue for future research, as it may help to indicate the extent to which the findings and conclusive messages of HRQoL research are affected by the Rasch-indicated psychometric shortcomings of the AS-20.

There are several limitations in this study that should be noted. Firstly, given that the study included a convenience sample of adults with strabismus who were recruited from a single tertiary eye care centre in South India, selection bias cannot be ruled out and the generalizability of the results may be compromised. Nevertheless, this should be balanced against the fact that a large sample size was utilized in the study. Future large scale studies need to be done to verify findings from this study. Secondly, in these analyses, only basic psychometric characteristics (i.e., reliability, unidimensionality) were considered, but features like test-retest reliability and responsiveness to change were not examined. Secondly, the version of AS-20 administered was based on participant’s language of preference, and their choices were not recorded. As such, the effect of different language versions on the analysis and potential DIF by language version could not be examined. Finally, the post-hoc solutions in this study can be considered “optimum” only in the current sample, and in other populations the results may look different. Further evaluation of the response category format of the instrument should be undertaken to examine the decision made in this study to rescore the categories of the items in the AS-20. Our participants answered the 5-point Likert scale, not a 3-point Likert scale; thus, we do not know the psychometric properties of the AS-20questionnaire using the 3-point Likert scale. Future studies are needed.A suggestion for a future study would be to administer the original and revised versions of the AS-20 scoring to a single cohort to compare their validity. If the findings are similar to the present study, then the revised scoring format consisting of three categories for the AS-20 can be recommended for future use.

## Conclusions

In conclusion, we have established that the Indian Rasch-scaled version of the AS-20 questionnaire comprising of two subscales is valid and reliable, and is linguistically and culturally suitable for use among Hindi and Telugu speaking patients in India. It provides a useful measurement tool to inform, design and evaluate the HRQoL in this patient population. The Indian versions of the AS-20 used in this study are easy to understand and quick to complete, and will serve as important tools to assess the HRQoL for adults with strabismus in most parts of India. A linear transformation of the raw scores from the two Rasch-revised AS-20 subscales can be used with confidence in parametric analyses for the Indian adult strabismic population. Further studies involving Rasch analysis would certainly contribute towards a better understanding of the dimensionality of the subscales of AS-20.

## Supporting Information

S1 Datasheet Excel data sheet of the 584 participants included in the study. (XLS)

S2 Datasheet Excel spreadsheets to convert raw scores to Rasch-scaled scores for the revised psychosocial subscale of AS20. (XLSX)

S3 Datasheet Excel spreadsheets to convert raw scores to Rasch-scaled scores for the revised function subscale of AS20. (XLSX)
